# Laparoscopic resection of a non-communicating uterine rudimentary horn using intra-operative indigocarmine injection: A case report

**DOI:** 10.1016/j.ijscr.2021.105743

**Published:** 2021-03-12

**Authors:** Toshiaki Takahashi, Satoshi Shiojima, Takashi Hamano, Hiroko Konno, Shunsuke Yamada

**Affiliations:** a Department of Pediatric Surgery, Seirei General Hospital, Hamamatsu, Japan; b Department of Gynecology, Seirei General Hospital, Hamamatsu, Japan; c Department of Colorectal Surgery, Seirei General Hospital, Hamamatsu, Japan

**Keywords:** UU, unicornuate uterus, RH, rudimentary horn, UUNCRH, unicornuate uterus with a non-communicating rudimentary horn, LIUICI, laparoscopic intra-uterus indigo carmine injection, MRI, magnetic resonance imaging, NSAIDs, non-steroidal anti-inflammatory drugs, Unicornuate uterus, Rudimentary horn, Laparoscopy, Indigo carmine

## Abstract

**Introduction and importance:**

An unicornuate uterus with a non-communicating rudimentary horn (UUNCRH) is a very rare uterine malformation which is difficult to diagnose and to decide the surgical plan. We aim to describe the case of pediatric UUNCRH patient and our operative technique of laparoscopic intra-uterus indigo carmine injection (LIUICI) to confirm that the rudimentary horn (RH) is non-communicating before the resection and review the relevant literature to ascertain the most appropriate treatment option in these patients.

**Case presentation:**

A 11-year-old girl who developed progressive severe abdominal pain and dysmenorrhea was referred to our hospital. Uterine malformation and right hematosalpinx was confirmed with magnetic resonance imaging (MRI). Pre-operative treatment with a gonadotropin-releasing hormone agonist enabled improvement in the symptoms. Laparoscopic exploration was scheduled. The right fallopian tube was resected laparoscopically and a 3Fr tube was inserted into its cut end. Indigo carmine injected in the RH through the tube. No leakage of indigo carmine was found from the vagina, indicating the diagnosis of the uterine malformation is an UUNCRH and we performed the resection of the RH safely.

**Clinical discussion:**

In pediatric patients transvaginal detailed examination is not easy to perform. Therefore, diagnostic and operative laparoscopy is critically important for the safe treatment. In addition, laparoscopic removal of a RH can be used to decrease the incidence of adhesions.

**Conclusion:**

We found LIUICI technique before the resection of the RH is safe, technically feasible and minimally invasive approach for pediatric UUNCRH patients.

## Highlights

• Unicornuate uterus with a non-communicating rudimentary horn is rare and difficult to diagnose.
• In pediatric patients, transvaginal detailed examination is not easy to perform.
• Laparoscopic intra-uterus indigo carmine injection is a simple, safe and valuable maneuver in non-communicating UUNCRH.

## 1 Introduction

The incidence of Mullerian duct anomalies range between 0.4–10% [1]. Mullerian duct anomaly is divided into four subgroups [2]. A unicornuate uterus (UU) is caused by a failure of development in one of the two Mullerian duct between the 7th and 8th week of gestation and frequently present with a rudimentary horn (RH) [3]. UU with a RH is a rare malformation, with a frequency of 0.06% [3]. Most RH are non-communicating [4] and the subgroup with presence of RH with a non-communicating cavity belongs to type II b [2].

UU with a non-communicating RH (UUNCRH) patients may present with dysmenorrhea, chronic pelvic pain and hematometra [5]. In the case of a blind non-communicating, cavitated RH with a functioning endometrium, cryptomenorrhoea may lead to dysmenorrhoea soon after menarche and result in haematometra [6]. Retrograde menstruation from a functioning RH through a patent ipsilateral Fallopian tube may result in the development of haematosalpinx [7].

In recent years, laparoscopy has become a viable alternative to laparotomy for management of the RH. However, it is difficult to ascertain preoperatively if the RH is communicating and to reveal the detail of the uterus anomaly in children, because transvaginal examination for young children is limited to perform in the outpatient clinic. Therefore, proper intra-operative diagnosis and surgical treatment are essential to prevent serious complications.

We aim to describe our operative technique of laparoscopic intra-uterus indigo carmine injection (LIUICI) to confirm that the rudimentary horn is non-communicating before the resection and review the relevant literature to ascertain the most appropriate treatment option in these UUNCRH patients.

## 2 Case presentation

We ensure our case report is compliant with the SCARE Guidelines 2020 [8].

### 2.1 Patients

**Fig. 1:**
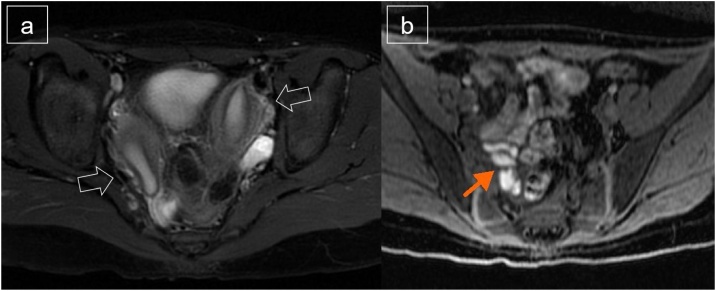
These Figures shows magnetic resonance imaging (MRI) findings. Uterine malformation (a: white arrows) and right hematosalpinx (b: orange arrow) was confirmed with this MRI.

A 11-year-old girl presented severe pain during her menstruation period which was only minimally relieved with medical management, non-steroidal anti-inflammatory drugs (NSAIDs). Uterine malformation and right hematosalpinx was confirmed with magnetic resonance imaging (MRI) (Fig. 1ab). Pre-operative treatment with a gonadotropin-releasing hormone agonist enabled improvement in the symptoms. Elective laparoscopic surgery was planned.

### 2.2 Operative procedures

**Fig. 2:**
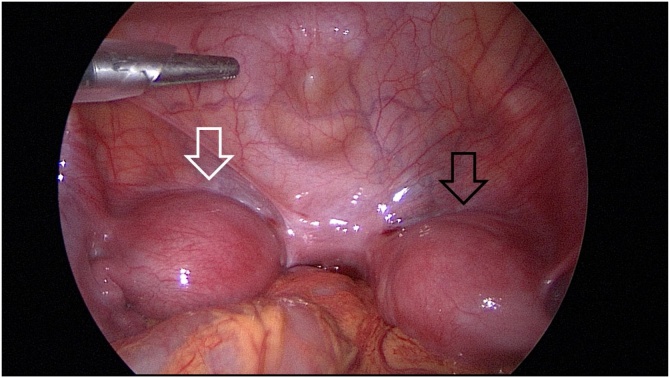
This figure shows laparoscopic findings. Laparoscopic exploration confirmed the presence of uterine malformation. White arrow shows the left side uterus which were thought to be normal. Black arrow shows the right side uterus.

**Fig. 3:**
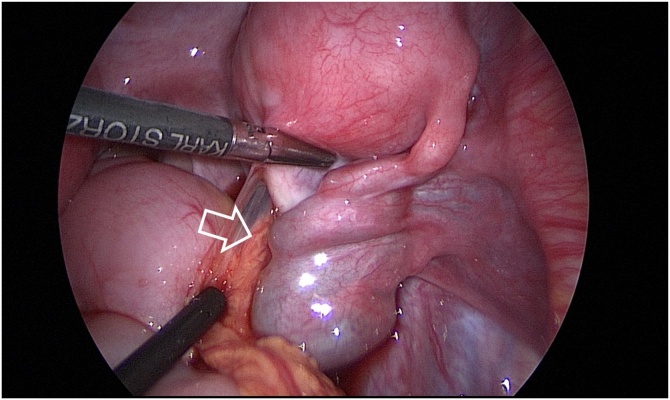
This figure shows the right hematosalpinx (white arrow).

**Fig. 4:**
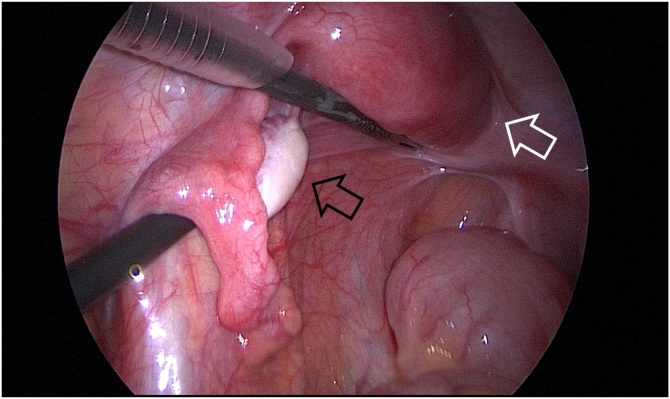
The left side uterus and ovary were thought to be normal. White arrow shows the left side uterus and black arrow shows the left side ovary.

**Fig. 5:**
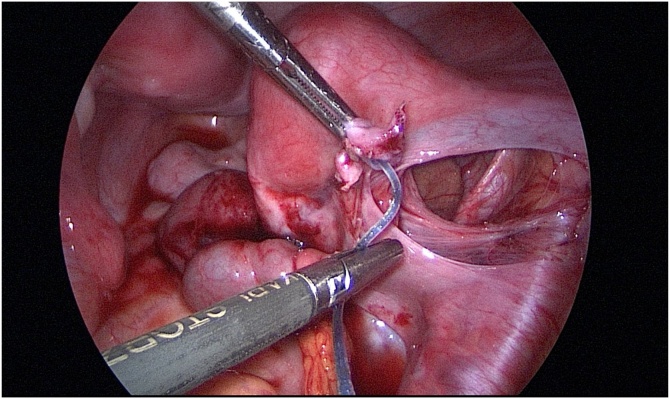
The right fallopian tube was resected laparoscopically and 3Fr feeding tube was inserted into its end cut off and injected indigo carmine in the rudimentary horn.

**Fig. 6:**
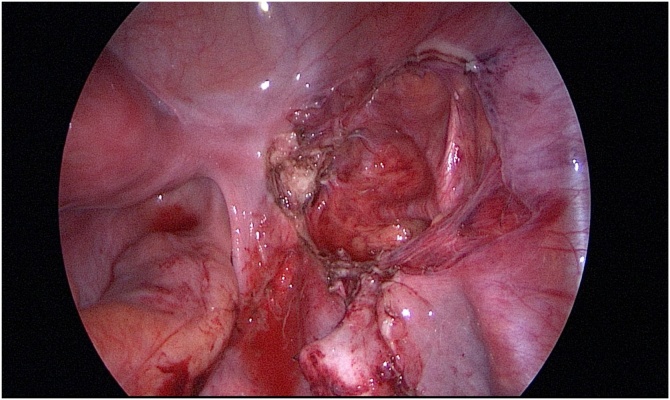
We performed the resection of the rudimentary horn safely.

**Fig. 7:**
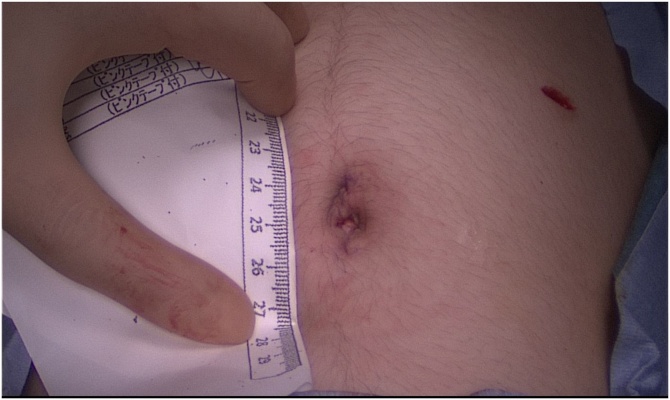
The scar was very small and the patients had good cosmetic results. This figure shows the umbilical scar.

During the operation, a patient was placed in a lithotomy position under general anesthesia. First, a 20 mm single vertical umbilical incision was made. The Lap-protector (alnote LapSingleTM, Applied Alfresa Pharma Coroperation, Japan) was then placed through the incision. After creating a pneumoperitoneum of 6–10 mmHg, a 5 mm, 30 degree laparoscope was inserted and three 5 mm trocars (EZ trocar®, Hakko Medical, Japan) were placed both sides of lower abdomen and left side of upper abdomen. Then, laparoscopic exploration confirmed the presence of uterine malformation (Fig. 2) and right hematosalpinx (Fig. 3). The left side uterus and ovary were thought to be normal (Fig. 4). It could not be identified whether the RH was communicating or non-communicating. The right fallopian tube was resected laparoscopically and 3Fr feeding tube was inserted into its end cut off and injected indigo carmine in the RH (Fig. 5). No leakage of indigo carmine was found from the vagina, indicating the diagnosis of the uterine malformation is an unicornuate uterus with a non-communicating RH (UUNCRH) and we performed the resection of the RH safely (Fig. 6). The scar was very small and the patients had good cosmetic results (Fig. 7).

## 3 Discussion

The incidence of congenital Mullerian duct anomalies has been reported as high as 3.2% [9,10]. A partial or complete lack of development of one Mullerian duct during weeks 7–8 of gestation may result in the formation of a UU [3]. Seventy-five to ninety percent of UU with RH are non-communicating [4]. Non-communicating cavitated RH are the most clinically significant as they are more likely to be associated with pelvic pain from haematometra or from endometriosis due to retrograde menstruation [11].

The UU with RH seems difficult to diagnose. The RH is often small and is not easy to palpate during bimanual examination [12]. The ultrasound, CT even MRI doesn’t always diagnose uterus anomaly properly [13]. Hysterosocpy are often useful tools in diagnosing this uterine anomaly and the transvaginal US may be used to evaluate the presence of a RH [14]. However, in pediatric patients transvaginal detailed examination is not easy to perform. Therefore, diagnostic and operative laparoscopy is critically important for the safe treatment. In addition, laparoscopic removal of a RH can be used to decrease the incidence of adhesions.

We report a case of a UU with a non-communicating RH presenting as severe dysmenorrhea. In our case, laparoscopic exploration confirmed the right hematosalpinx and the right fallopian tube was resected laparoscopically. Then, we inserted 3Fr feeding tube into the RH and injected indigo carmine. We confirmed that the diagnosis of the uterine malformation is an UUNCRH. Owing to this procedure, we performed the resection of the RH safely. Furthermore, the scar was very small and the patients had good cosmetic results.

## 4 Conclusion

The LIUICI technique was valuable to exclude a communicating uterine horn before minimal-invasive resection in patients with UUNCRH. The technique is simple and safe to perform laparoscopically.

## Declaration of Competing Interest

The authors declare that they have no competing interests.

## Sources of funding

The authors declare that they received no funding support for this study.

## Ethical approval

Not applicable.

## Consent

Written informed consent was obtained from the patient for publication of this case report and accompanying images. A copy of the written consent is available for review by the Editor-in-Chief of this journal on request.

## Author contribution

TT acquired the data and drafted the manuscript. TT, SS, HK and SY performed the operations. All other authors attended the patient postoperatively. All authors read and approved the final manuscript.

## Registration of research studies

Not applicable.

## Guarantor

Not applicable.

## Provenance and peer review

Not commissioned, externally peer-reviewed.
